# QTL mapping of egg albumen quality in egg layers

**DOI:** 10.1186/1297-9686-45-31

**Published:** 2013-08-16

**Authors:** Mervi Honkatukia, Maria Tuiskula-Haavisto, Jesus Arango, Jonna Tabell, Matthias Schmutz, Rudolf Preisinger, Johanna Vilkki

**Affiliations:** 1 MTT Biotechnology and Food Research, Jokioinen 31600, Finland; 2 Lohmann Tierzucht GmbH, Cuxhaven, Germany; 3 Hy-Line International, P.O. Box 310, Dallas Center, IA 50063, USA

## Abstract

**Background:**

A fresh, good quality egg has a firm and gelatinous albumen that anchors the yolk and restricts growth of microbiological pathogens. As the egg ages, the gel-like structure collapses, resulting in thin and runny albumen. Occasionally thin albumen is found in a fresh egg, giving the impression of a low quality product. A mapping population consisting of 1599 F_2_ hens from a cross between White Rock and Rhode Island Red lines was set up, to identify loci controlling albumen quality. The phenotype for albumen quality was evaluated by albumen height and in Haugh units (HU) measured on three consecutive eggs from each F_2_ hen at the age of 40 weeks. For the fine-mapping analysis, albumen height and HU were used simultaneously to eliminate contribution of the egg size to the phenotype.

**Results:**

Linkage analysis in a small population of seven half-sib families (668 F_2_) with 162 microsatellite markers spread across 27 chromosomes revealed two genome-wide significant regions with additive effects for HU on chromosomes 7 and Z. In addition, two putative genome-wide quantitative trait loci (QTL) regions were identified on chromosomes 4 and 26. The QTL effects ranged from 2 to 4% of the phenotypic variance. The genome-wide significant QTL regions on chromosomes 7 and Z were selected for fine-mapping in the full set composed of 16 half-sib families. In addition, their existence was confirmed by an association analysis in an independent commercial Hy-Line pure line.

**Conclusions:**

We identified four chicken genomic regions that affect albumen quality. Our results also suggest that genes that affect albumen quality act both directly and indirectly through several different mechanisms. For instance, the QTL regions on both fine-mapped chromosomes 7 and Z overlapped with a previously reported QTL for eggshell quality, indicating that eggshell membranes may play a role in albumen quality.

## Background

Eggs for the table egg market should be microbiologically safe and look good. The number of eggs for processing egg products has increased during recent years, emphasizing the importance of high quality and good processing properties. Good quality albumen has a firm jelly-like structure that keeps the yolk in the center of the egg. Albumen quality starts to degenerate immediately after the egg is laid and thinning is a natural process during storage. For breeding purposes, albumen quality is measured in Haugh units (HU), expressed as a function of egg weight and the albumen height (AH) of a broken egg [1]. The egg industry is particularly interested in functional properties such as coagulation and foaming, which makes quality assessment complex [2].

Genetic background can explain, in part, differences in albumen quality among individuals and breeds [3]. The average heritability of albumen quality is moderate, reaching 0.30 [4]. It has also been demonstrated that sires have a higher influence on the heritability of AH and HU than dams, which indicates a sex-linked effect [4]. Among environmental factors, management and egg storage conditions have a substantial impact on maintaining albumen height, while feed composition has only a minor effect on albumen quality [2,5]. Furthermore, viruses in the reproductive tract may lead to the production of watery white eggs and extremely poor quality albumen [5].

Various causes of albumen deterioration have been suggested. Imperfections can emerge during the early formation of albumen in the reproductive tract but also after oviposition. A potential explanation for the decline in quality with time is linked with eggshell and membrane traits. An intact eggshell with good inner and outer membrane structures plays an important role in albumen quality, particularly during storage, preventing evaporation and escape of metabolic gases through the shell pores. CO_2_ leak is known to change albumen pH towards alkaline values [6].

Albumen quality, among other egg quality and production traits, is a typical quantitative trait that has been studied by QTL mapping. Nevertheless, among the available data on chicken QTL, there are relatively few QTL that affect albumen quality [7]. In the ChickenQTLdb, 16 distinct QTL locations are associated with HU, AH or albumen weight (AW). In the database, QTL regions that influence albumen quality are located on chromosome 1 (HU between positions 48.17 and 53.13 Mb; AH and HU between 90.35 and 123.03 Mb) [8,9], chromosome 2 (HU between 5.31 and15.36 and between 31.23 and 38.97 Mb and AH between 80.69 and 104.34 Mb) [9,10], chromosome 3 (AW at position 106.44 Mb) [11] and chromosome 4 (AH and AW at 9.45 Mb, AW between 62.18 and 75.89 and AW at ~80 Mb) [11,12]. In addition, genome-wide association studies revealed significant SNP associations on chromosomes 1, 3, 5, 18, 19, 23 and Z with early or late AH [13] and other interesting associations have been reported on chromosomes 7, 8, 9, 14, 20 and 24 [14]. These studies have revealed that several overlapping genomic regions are involved in various egg quality traits, but the relationships between these regions have not yet been detailed at the biological level. Simple text-based searches in the chicken genome [15] for genes associated with egg white yielded 20 hits, eight of which referred to known QTL regions, the remainder being annotated albumen related genes, such as *LYZ* (*lysozyme*, chr 1: 37.29 Mb)*, LYG2* (*lysozyme G-like 2,* chr 1: 136.64 Mb), *PRL* (*prolactin*, chr 2: 59.7 Mb)*, SERPINB6* (*serine (or cysteine) peptidase inhibitor, clade B, member 6e*, chr 2: 68.85 Mb), *CALB1* (*calbindin 1, 28kDa*, chr 2: 129.15 Mb), *CST3* (*cystatin C*, chr 3: 16.49 Mb)*, SPP1* (*secreted phosphoprotein 1,* chr 4: 47.10 Mb)*, MUC6* (*mucin 6, oligomeric mucus/gel-formin,* chr 5: 16.121 Mb)*, MUC5B* (*ovomucin, alpha subunit*, chr 5: 15.95 Mb) and *TF* (*transferrin*, chr 9: 5.62 Mb). Although studies on the egg albumen proteome have significantly expanded the list of identified albumen proteins [16,17], and the mechanisms involved in egg white thinning have been studied at the protein level [18], no causal variations in albumen genes have yet been identified.

In this study, we used an F_2_ intercross between the two egg-layer lines, White Rock and Rhode Island Red, in order to identify QTL that affect albumen quality. Mapping was performed in three steps: (1) identification of QTL in a small F_2_ population, (2) fine-mapping of these QTL in a larger F_2_ population, and (3) verification of the QTL in a commercial line, Hy-Line. We also investigated possible links between albumen quality and eggshell properties based on overlapping QTL results in the ChickenQTLdb (on the chicken genome build WASHUC2).

## Methods

### Mapping populations

For mapping, an F_2_ population was created between two commercial egg-layer lines from Lohmann Tierzucht i.e. Rhode Island Red and White Rock. The crossed lines differed in albumen quality; the average HU was 56.31 for Rhode Island Red and 69.29 for White Rock (Table 1). Practical management was similar to that used in previous QTL studies [19]. The full mapping population was made up by reciprocal crosses between 14 Rhode Island Red individuals (six males and eight females) and 15 White Rock individuals (six males and nine females). The F_1_ generation consisted of 16 males and 96 females, leading to 16 half-sib families. A genome scan was performed with 162 microsatellite markers spread across 27 of the 39 chicken chromosome pairs using a subset of seven half-sib families with 668 F_2_ individuals. The full F_2_ mapping population of 1599 individuals was used for fine-mapping.

**Table 1:** **Descriptive statistics of the phenotypes analyzed in the study for the F**_**2 **_**and Hy-Line populations**

**Population**	**N**	**Trait and unit**	**Average**	**Standard deviation**	**Min-Max**
Rhode island red	84	AH, after one week storage, mm	5.8	0.39	4.5-7.3
White rock	82	AH, after one week storage, mm	6.1	0.42	4.6-7.7
F_2_	1599	HU, Haugh units	85.0	6.89	59.6-105.8
		AH, mm	7.3	1.15	2.9-11.8
Hy-Line	299	AH early, mm	8.4	0.44	7.32-9.57
Hy-Line	221	AH late, mm	8.0	0.55	5.73-9.37

The phenotypes for parental lines (Rhode Island Red and White Rock) are breeding values for AH after one week storage.

In order to reanalyze the QTL regions, a commercial egg laying chicken population (Hy-Line) was used. The Hy-Line population consisted of 290 males belonging to paternal half-sib families (3.5 males per half-sib family).

### Phenotypes

Albumen quality was evaluated in the F_2_ mapping population for three consecutive eggs, each within 24 h of laying, for each hen at the age of 40 weeks. To measure albumen quality, the egg was weighed, broken on a glass plate and the height of the thick albumen (AH) was measured with a micrometer. AH was transformed into Haugh units (HU) with a standardized function of the AH and egg weight, and corrected with a constant [1]. In the whole-genome scan, only the corrected HU estimations were used, but both AH and HU estimations were used to exclude contribution of the egg weight to the phenotype and to make the different datasets comparable. In the commercial Hy-Line population, albumen quality was assessed by AH twice during the production period i.e. at 26 weeks of age (= early) and again at 42 to 44 weeks of age (= late). The phenotypes are presented as sire-daughter averages and are described in Table 1.

### Genotyping

DNA preparation and genotyping of microsatellite markers were carried out as described by Tuiskula-Haavisto et al. [20], who also reported marker maps and information contents along the chromosomes. For chromosome 7, genotyping of the entire F_2_ mapping population was performed with five microsatellite markers covering 96 cM (MCW361, ADL326, MCW183, MCW236 and ADL315, see Table 2), three of which (ADL326, MCW183 and MCW236) were also genotyped in the Hy-Line population. For fine-mapping on chromosome Z, a selected a set of SNP markers covering the QTL region (MCW258-MCW241) were used instead of microsatellite markers. An Illumina BeadXpress [21] reader was used to genotype multiplex SNP in both mapping populations, as reported in [22]. In the F_2_ population, 20 informative SNP markers were selected for linkage analysis of the QTL regions (Table 3). In the Hy-Line population, 12 SNP markers were genotyped on chromosome Z, of which six were included in the QTL region (Table 3). Because different SNP segregated in the different populations, the marker sets analyzed were not completely identical in the different populations.

**Table 2:** **Best results from the initial QTL mapping based on 668 F**_**2 **_**individuals and HU**

**Chr**	**Marker map**	**Markers flanking the QTL and their genomic positions**	**F-ratios and corresponding boundaries for p-level (CI length in brackets)**	**Additive effect (SE)**	**Dominance effect (SE)**	**R**^**2**^
**7**	MCW361*-(1)-***ADL326***-(54)-***MCW183***-(31)*-**MCW236***-(10)-*ADL0315	MCW183-MCW236 (24.25-29.72 Mb)	F = 8.32 (T01 8.76; T05 7.17) (CI = 57 cM)	14.0 (3.45)	1.83 (5.32)	0.04
**26**	MCW355*-(13)-*MCW285*-(35)-*ADL885	MCW285-ADL285 (2.50-4.91 Mb)	F = 6.04 (T05 7.01; T10 4.61) (CI = 49 cM)	12.51 (5.24)	27.22 (11.70)	0.02
**4**	MCW47*-(43)-*MCW5*-(37)-*ADL266*-(15)-*LEI94*-(6)-*MCW284*-(12)-*ADL331*-(11)-*MCW170*-(5)-*MCW180*-(12)-*MCW122*-(6)-*LEI119*-(15)-*MCW99*-(9)-*LEI73	MCW122-LEI119 (76.43-80.94 Mb)	F = 6.49 (T05 8.02; T10 5.02) (CI = 146 cM)	12.83 (3.66)	4.84 (5.74)	0.02
**Z**	ADL117*-(22)-*MCW331*-(12)-*MCW55*-(6)-*MCW258*-(28)-*LEI171*-(4)-*ADL201*-(6)-*MCW241*-(5)-*LEI229*-(1)-*MCW154*-(1)-*MCW246*-(5)-*LEI254*-(1)-*MCW294*-(3)-*ROS117*-(3)-*LEI111*-(1)-*LEI144*-(1)-*LEI121*-(28)-*LEI75*-(5)-*MCW269	MCW258-MCW241 (21.40-34.26 Mb)	F = 39.20 (T0001 19.03; T00119.98; T01 9.55; T05 6.52)	15.28 (2.44)	NA	0.02

Chr chromosome number; distances between markers (in parentheses) are based on Haldane’s mapping function; markers genotyped in the commercial line are indicated in bold; the markers flanking the QTL and their locations are indicated in Mb (WASHUC2), followed by F-ratios and corresponding boundaries for p-values, where T0001 to T10 represent p-level thresholds from 0.0001 to 0.05 (boundary for genome-wide mapping, T10 is a suggestive level QTL ); CI confidence interval, SE standard error; R^2^ = proportion of phenotypic variance explained by QTL for the additive effect.

**Table 3:** Fine-mapping results within different populations using different mapping methods and marker compositions

**Chr**	**Pop**	**N**	**Method**	**Marker composition and genomic position**	**Markers flanking the QTL/associated marker**	**Trait**	**p-value**	**Effect**	**R**^**2**^
**Z**	F_2_	1599	QTL linkage mapping by custom made program	rs16765819(29.091.210)-rs14687314(30.458.261)- rs14691747(30.806.242)-rs13799822(31.307.747)- rs14762832(31.855.782)-rs16766794(31.956.374)- rs16766752(32.044.710)-rs16766685(32.277.106)- rs16766334(33.023.048)-rs14761691(33.305.926)- rs13795456(33.672.729)-rs14761341(33.749.560)- rs14761196(33.997.081)-rs16767662(34.996.569)- rs16110154(35.510.105)-rs16767980(36.026.140)- rs16110443(36.236.898)-rs16111109(36.960.473)- rs16132282(39.449.000)- rs16684439(42.958.949)	rs14761341-rs16767662	HU	< 0.0001	12.57 (1.77)	0.03
					(at 33.75-35.00 Mb)				
					peak at position of rs14761196	AH	< 0.0001	1.87 (0.30)	0.02
	Hy-Line	290	PLINK	rs14700116(1.119.301)-rs16741325(1.411.493)- rs14067906(1.567.603) rs16726302(1.876.555)- rs14067572(1.991.047)-rs14067220(2.364.494)- rs16765819(29.091.210)-rs13816749(30.040.859)- rs13795687(32.123.345)-rs14761341(33.749.560)- rs14761196(33.997.081) rs14763225(34.275.437)	rs16785819 at 29.091.210	AH-early	0.03	3.86 (0.04)	0.06
**7**	F_2_	1599	QTL linkage mapping by GridQTL	MCW361-(1)- ADL326-(54)-MCW183-(31)- MCW236-(10)-ADL0315	flanked by MCW183-MCW236 (24.52-29.72 Mb)	AH	< 0.01	2.26	0.02
							LOD 6.90	(0.40)	
					(CI = 40–75 cM)				
						HU	<0.01	14.16	0.02
							LOD 6.83	(2.52)	
	Hy-Line	90-290	Single markers associations	ADL326 alias anlyrin repeat and SOCS box containing 18 (5.152.776);	ADL326	AH-early	0.0046	NA	NA
				MCW183 (24.245.453); MCW236 (29.724.317)		AH-late	0.0024	NA	NA

Chr chromosome number, Pop population, N number of individuals in the population; statistically the most probable positions of the QTL are indicated according to the positions of the flanking SNP markers in Mb (on WASHUC2); R^2^ = proportion of phenotypic variance of the effect.

### Statistical analysis

Marker maps were constructed with CRI-MAP [23] using procedures TWOPOINT, BUILD, FLIPS and CHROMPIC. QTL analyses were performed using the least squares method via the web-based GridQTL software [24]. Significance thresholds for QTL analysis were determined empirically by permutation, and confidence intervals were based on bootstrapping. The length of the chromosomes was taken into account when defining the significance thresholds. More information on the models used and how the significance levels and confidence intervals were derived is available in [19]. Fine-mapping in the F_2_ population used the same software for the chromosome 7 data, whereas a custom-made regression program [22] was applied to the chromosome Z data. The significance levels for the linkage analysis were obtained using a permutation procedure as explained in [19].

For the commercial Hy-Line, microsatellite marker associations were tested with a non-parametric Kruskal*–*Wallis test, because the genotyping data comprised a single generation and linkage analysis could not be applied. Let μ_ij_ be the trait mean for genotype *j* in marker *i,* and *i* = 1,…,3. The total amount of different genotype groups per marker is N_i_ and depends on the marker, so that *j* = 1,…,N_i_. The null hypothesis to be tested is then H_0i_: μ_i1_ = μ_i2_ =…= μ_iNi_ versus: H_1i_: μ_ik_ ≠ μ_il_ for at least one pair of genotypes *k* ≠ l and *k, l* ≤ N_*i*_.

Chromosome Z was analyzed with PLINK [25]. Data were checked for genotyping quality and Hardy-Weinberg equilibrium (none of the markers were excluded) before analysis. Basic association testing for quantitative traits and adjustment for multiple comparisons were used.

## Results

The whole-genome scan using low-density marker maps and seven half-sib families from the mapping population (668 of 1599 F_2_ individuals) detected four QTL regions that affect egg white quality (HU) (Table 2). A genome-wide significant QTL (p < 0.05) was found on chromosome 7 between microsatellite markers MCW183 and MCW236 (at the genomic positions 24.24 Mb and 29.72 Mb, respectively). The additive effect of the locus was 14.0 HU and accounted for 2% of the phenotypic variance. A highly significant QTL (p < 0.0001) was detected on chromosome Z between microsatellite markers MCW258 and MCW241 (at 21.40 Mb and 34.26 Mb, respectively) [see Additional file 1].The effect of this locus was 15.28 HU. A suggestive (5% chromosome-wide) QTL was found on chromosome 4, between markers MCW122 and LEI119 (76.43 and 80.94 Mb, respectively), which explained 2% of the trait variation, the effect being 12.83 HU. Similarly, a putative QTL was detected on chromosome 26 between markers MCW285 and ADL285 (2.50 and 4.91 Mb, respectively). In contrast to the other identified QTL, this QTL displayed a large dominance effect.

**Figure 1:**
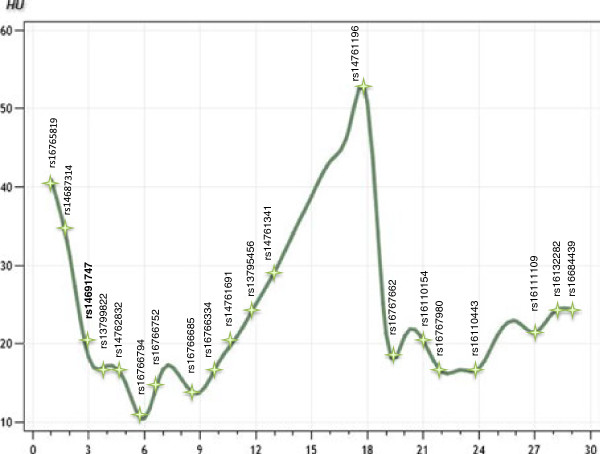
**QTL graph for chromosome Z.** The analysis focused on the QTL region detected previously (chromosome location in cM); the full F_2_ mapping population (1599 individuals), SNP markers (named according to the rs-code) and HU phenotype data were used; the highest F-value for the QTL (50.73) co-located with SNP marker rs14761196.

Various strategies were used to explore the data in greater detail. Both AH and HU measurements were used in order to eliminate contribution of the egg weight to the phenotype and to allow comparison of different datasets. All 16 half-sib families were genotyped with five microsatellite markers on chromosome 7. Linkage analysis of this full F_2_ mapping population increased the resolution and significance of the QTL, reaching the 1% genome-wide significance level (Table 3). However, the confidence intervals remained large because of the low-coverage map. The QTL on chromosome Z was fine-mapped using the F_2_ population and linkage mapping with 20 SNP markers covering the QTL region. The microsatellite markers originally used were excluded from the analysis in order to avoid bias from heterogeneous information content arising from the two types of markers. Increasing the sample size (to the full mapping population) made it possible to focus on the region containing the QTL in the SNP analysis. The QTL affecting both AH and HU was located in a region between 33.75 and 34.99 Mb, flanked by the markers rs14761341 and rs16767662 (Figure 1). The QTL peak co-located with the SNP marker rs14761196. Results for AH and HU were consistent, the only difference being that the result for HU was more significant than for AH (F = 50.73 vs. F = 40.11). We did not find any evidence for a QTL involved in egg weight but the results suggested the presence of QTL that affect eggshell strength (data not shown).

The commercial Hy-Line population was used to confirm the most promising QTL regions, on chromosomes 7 and Z. On chromosome 7, microsatellite ADL326 at position 5.15 Mb was significantly associated with both early and late AH (p = 0.0046 and 0.0024, respectively) between different genotypes [see Additional file 2]. Three segregating alleles formed seven genotype classes in the Hy-Line population, with on average 11.5 individuals in each group. On chromosome Z, marker rs16765819 at position 29.09 Mb was significantly associated with early and late AH (p = 0.0003 and 0.004, respectively). This locus was located at the distal end of the QTL region identified with the F_2_ population. Although rs16765819 was the outermost studied marker, it was significant in both populations.

## Discussion

We used an F_2_ intercross between the two egg-layer lines, White Rock and Rhode Island Red, to identify QTL associated with albumen quality and detected four genomic regions on chromosomes 4, 7, 26 and Z. The QTL on chromosome 26 has not been reported before. Evidence for the QTL on chromosome 4 (between positions 76.43 and 80.94 Mb) is indirectly supported by the QTL reported for albumen weight in two other studies, respectively between positions 62.18 and 75.89 Mb in [12] and at 80 Mb in [11].

The QTL on chromosome 7 between positions 24.24 Mb and 29.72 Mb co-located with the HU40 association reported by Liu et al. [14], using two experimental egg-type lines, White Leghorn and dwarf brown layers. This same region overlapped with a QTL that affects eggshell strength between positions 24.25 and 31.83 Mb identified by Sasaki et al. [26]. In addition to these, Abasht et al. [13] detected a significant 3-SNP window for early shell quality at a position between 25.1 and 26.2 Mb. Furthermore, Liu et al. [14] identified putative associations with eggshell thickness (EST40) between 27.47 and 29.06 Mb. It should be noted that all these studies used brown layer lines but belonging to different breeds [13,14,26].

Previously, it was shown that the QTL for eggshell thickness [26] and shell strength [19] at position 35 Mb overlapped on chromosome Z. Our results reveal that the genomic region, around 29–35 Mb, also influences albumen quality. The fact that this region is on chicken chromosome Z agrees with the traditional knowledge that sires have a larger effect than dams on albumen quality [27].

A positive correlation between albumen and eggshell qualities has been reported [4]. In addition, several studies have suggested that QTL associated with albumen quality overlap with a shell-related QTL, which cannot be considered as pure coincidence. Two hypotheses can be proposed i.e. either genes common to both albumen quality and eggshell quality exist in these chromosomal regions or the shell-related QTL has pleiotropic effects on albumen quality (or vice versa). One possible common factor connecting albumen quality and shell character is the eggshell membranes, which are waterproof barriers against metabolic gases and water. The outer membrane i.e. cuticle, represents the outermost permeability control [28]. Barrier properties depend on the shell pores and distribution of the cuticle over the surface of the egg. The cuticle membrane can be partially or totally absent [28]. Because egg white quality deteriorates during storage, the barrier properties of shell membranes are likely to affect egg white quality significantly, at least after oviposition. According to one theory, egg white thinning is caused by a change in pH in the albumen e.g. [18]. This alteration arises by the evaporation of gases through the shell. Moreover, the cuticle contributes to eggshell thickness [29] and thus the recurrent co-locations of QTL associated with eggshell quality and albumen quality might be explained by the key roles played by the eggshell and membranes to prevent albumen deterioration. According to another theory, multifunctional genes control egg traits such as albumen and shell properties. This multipurpose functioning hypothesis is supported, for instance, by the results of Hincke et al. [30] and Jonchere et al. [31], who both showed that some egg white proteins are found also in the shell (or shell membranes). Nevertheless, it seems that egg albumen quality and shell traits are connected at the gene level.

Many promising candidate genes for albumen quality are present in the QTL regions detected. Some of the most relevant candidates from each QTL region are discussed below. The region detected on chromosome 7 between positions 23 and 30 Mb seems to be important for egg quality traits and includes a cluster of cell shape and adhesion genes, such as *ITGB3* (*integrin, beta 3 (platelet glycoprotein IIIa, antigen CD61),* 23.38 Mb), *DES* (*desmin,* 23.73 Mb), *VIL1* (*villin 1,* 24.09 Mb), TNS4 (*tensin 4,* 24.09 Mb), ITGB5 *(integrin, beta 5*, 29.46 Mb) and *MUC13* (*mucin 13, cell surface associated,* 29.51 Mb). In particular, *MUC13* is a very attractive candidate for controlling albumen quality because the ovomucin protein, consisting of mucin subunits, is believed to be responsible for the gel-like structure of the fresh albumen [16,17,28,32,33].

The QTL peak on chromosome Z co-located with SNP marker rs14761196 at position 33.99 Mb that lies within an uncharacterized gene, *KIAA1797*. This gene is expressed widely in reproductive and secretory tissues. Both the independent commercial Hy-Line population and the F_2_ mapping population showed a very significant association with marker rs16765819 at position 29.09 Mb on chromosome Z that is located in the border region of the QTL. Marker rs16765819 is located near to the *PTPRD* (*protein tyrosine phosphatase, receptor type, D*). Other putative candidate genes near the QTL peak are, for instance, *GCNT1* (*glucosaminyl (N-acetyl) transferase 1, core 2 36*.9 Mb), which plays a role in mucin biosynthesis (KEGG entry 427260) and *UBQLN1* (*ubiquilin 1,* 39.4 Mb), which is required during protein degradation.

The number of SNP on chromosome Z differed between the populations studied. Within the QTL region fine-mapped on chromosome Z, six SNP were informative in the Hy-Line population, but only three of these were common in the two mapping populations. Although this complicates comparisons and interpretation of the results between the populations, our results were in line and were unambiguous for the commercial line.

Clusters of significantly associated markers, as on chromosomes 7 and Z, could be due either to a high number of causative polymorphisms or to a single causative polymorphism in the region with a high level of LD [33]. The effect of each associated SNP in the cluster might have a small effect, but within the region, the haplotypes could have a large overall effect on the trait studied. Many results suggest that accumulation of variations at the same locus is a relatively common mechanism [33].

## Conclusions

We identified four genomic regions that affect albumen quality in chicken of which those on chromosomes 4 and 26 are novel. The QTL on chromosomes 7 and Z overlapped with previously identified QTL for shell quality, which suggests the existence of possible common factors for both albumen and shell quality. The results of this study are congruent with the general assumption that multi-factorial causes are involved in egg albumen thinning. The genes that control albumen quality are diverse and act either directly or indirectly via different mechanisms. Egg white thinning is an intricate process that can take place anywhere during the process, starting from albumen formation in the magnum to egg storage after oviposition.

## Competing interests

The authors declare that they have no competing interests.

## Authors’ contributions

MH designed the genotyping work, participated in the statistical analysis (QTL mapping and association analyses) and wrote the manuscript. JT performed the genotyping. MT-H contributed to the design of the study and to data collection and analyses. JA, MS and RP contributed to the design of the study, provided phenotypic data and animal samples. JV supervised the study and edited the manuscript. All authors read and approved the final manuscript*.*

## Supplementary Material

Additional file 1 **HU QTL results within 668 F2 on chromosome 7 and Z during the initial scan with the low-coverage marker map.** Short description: QTL on chromosomes 7 and Z derived by a multi-marker regression method (Y-axis = F-ratio, X-axis = location in cM). Click here for file

Additional file 2 **Significant genotype-trait association plots obtained from the commercial line (Hy-Line) with different markers and phenotypes.** Short description: Marker association test in commercial line with a non-parametric Kruskal-Wallis test. Click here for file
