# Supplementary figures and images for: QTL mapping of egg albumen quality in egg layers
Source: Genet Sel Evol. 2013 Aug 16;45(1):31. doi: 10.1186/1297-9686-45-31 (PMC3847062; doi:10.1186/1297-9686-45-31)

**HU**

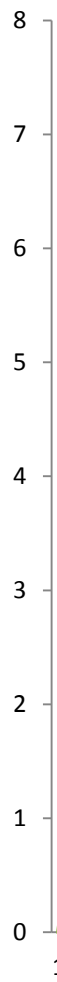

1

20

39

58

77

96

**Chr 7**

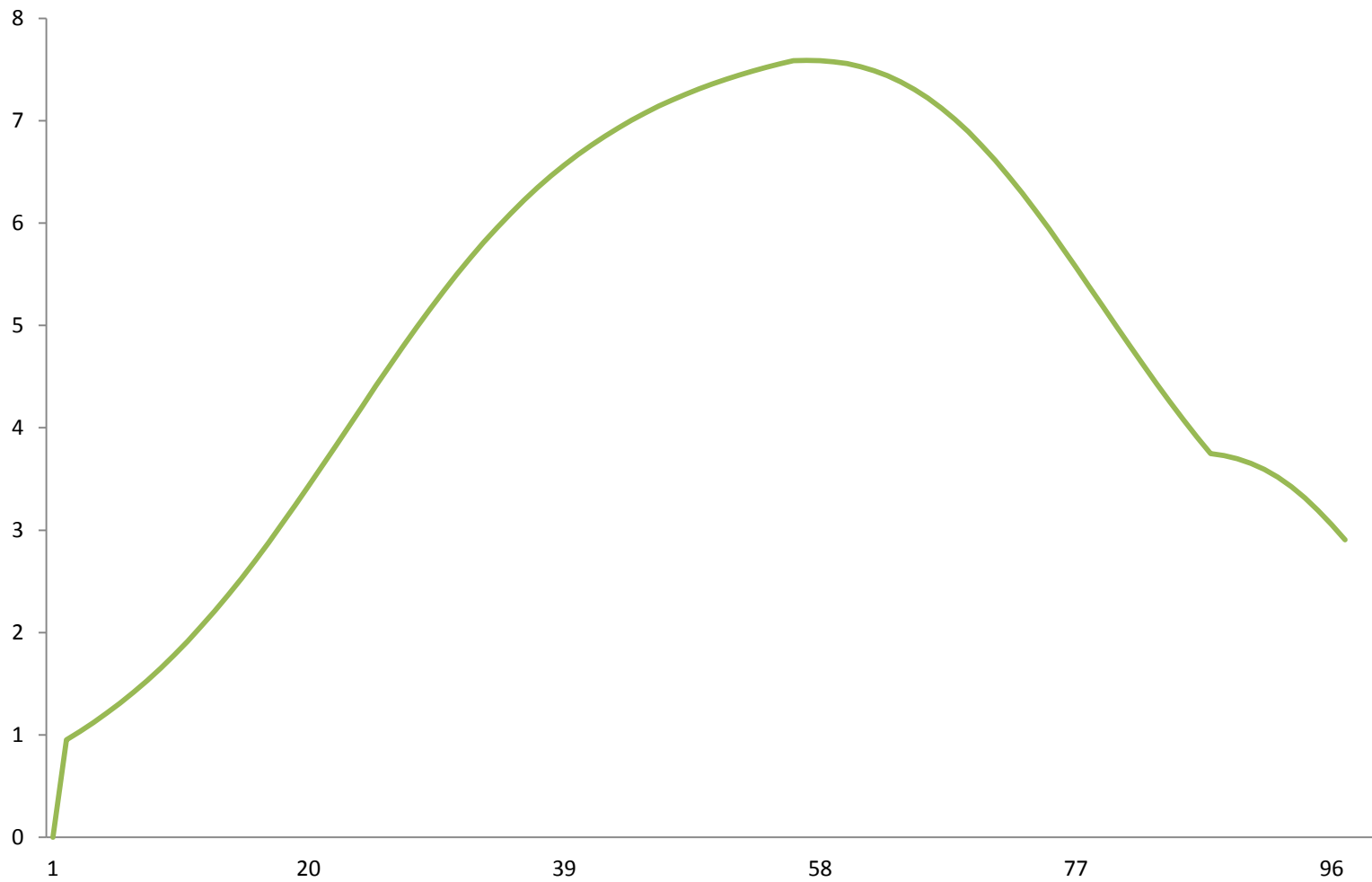

**HU**

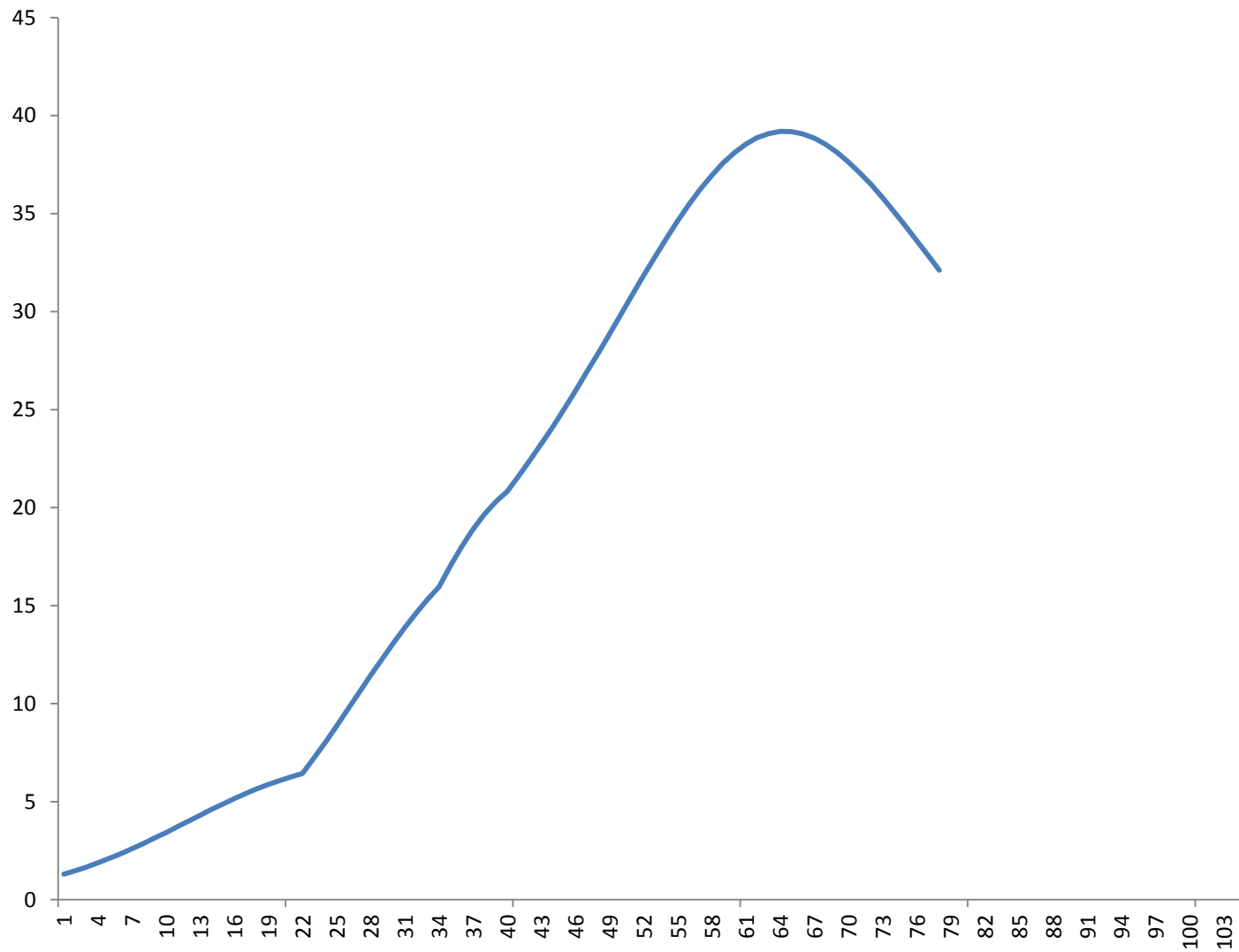

**Chr Z**

Supplement: Additional file 1 — HU QTL results within 668 F2 on chromosome 7 and Z during the initial scan with the low-coverage marker map. Short description: QTL on chromosomes 7 and Z derived by a multi-marker regression method (Y-axis = F-ratio, X-axis = location in cM). [file 1297-9686-45-31-S1.pdf]
