# Supplementary material for: QTL mapping of egg albumen quality in egg layers
Source: Genet Sel Evol. 2013 Aug 16;45(1):31. doi: 10.1186/1297-9686-45-31 (PMC3847062; doi:10.1186/1297-9686-45-31)
Supplement: Additional file 2 — Significant genotype-trait association plots obtained from the commercial line (Hy-Line) with different markers and phenotypes. Short description: Marker association test in commercial line with a non-parametric Kruskal-Wallis test. [file 1297-9686-45-31-S2.pdf]

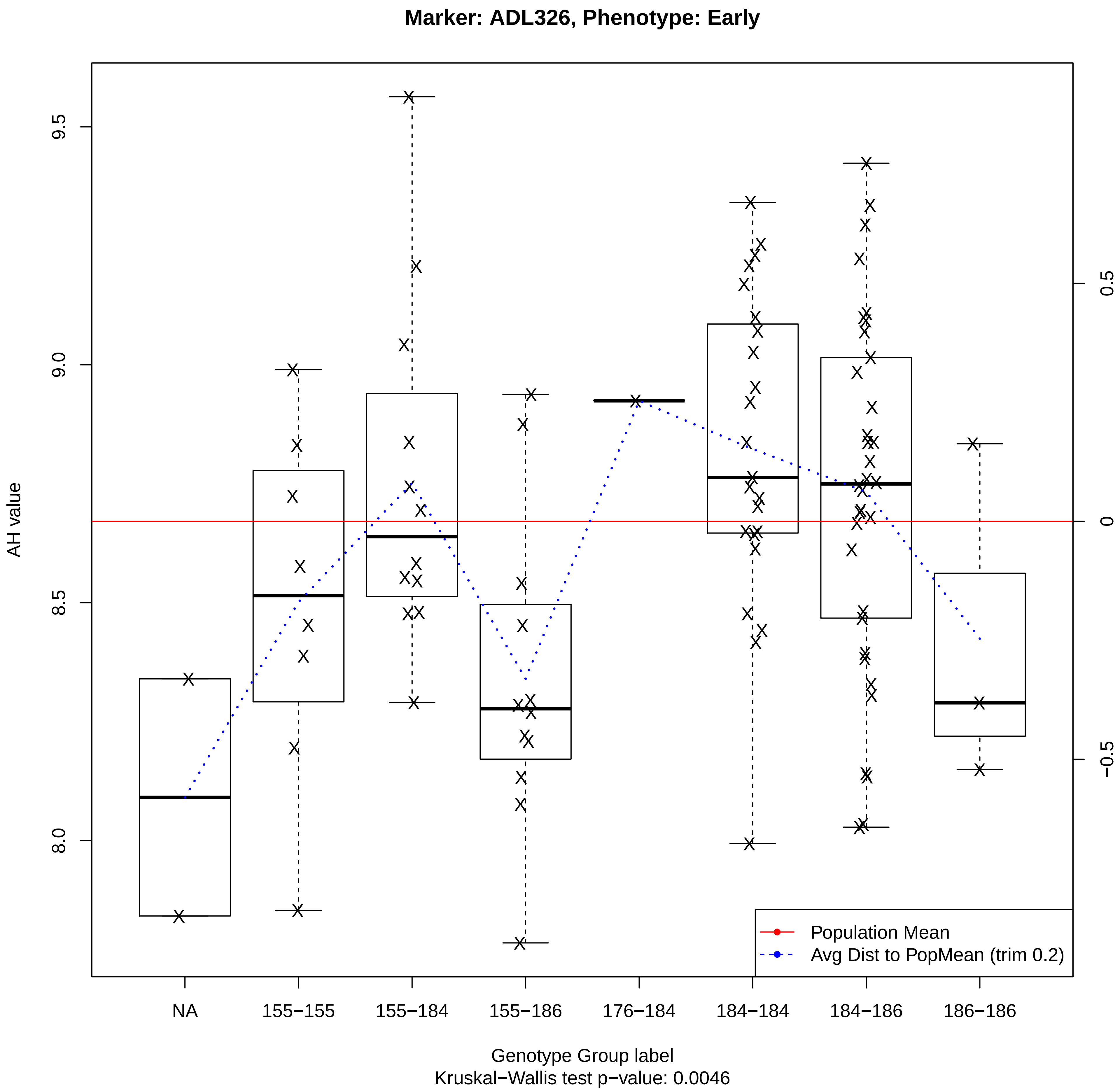

Marker: ADL326, Phenotype: Early
AH value
9.5
9.0
8.5
8.0
0.5
0
−0.5
Population Mean
Avg Dist to PopMean (trim 0.2)
NA
155−155
155−184
155−186
176−184
184−184
184−186
186−186
Genotype Group label
Kruskal−Wallis test p−value: 0.0046

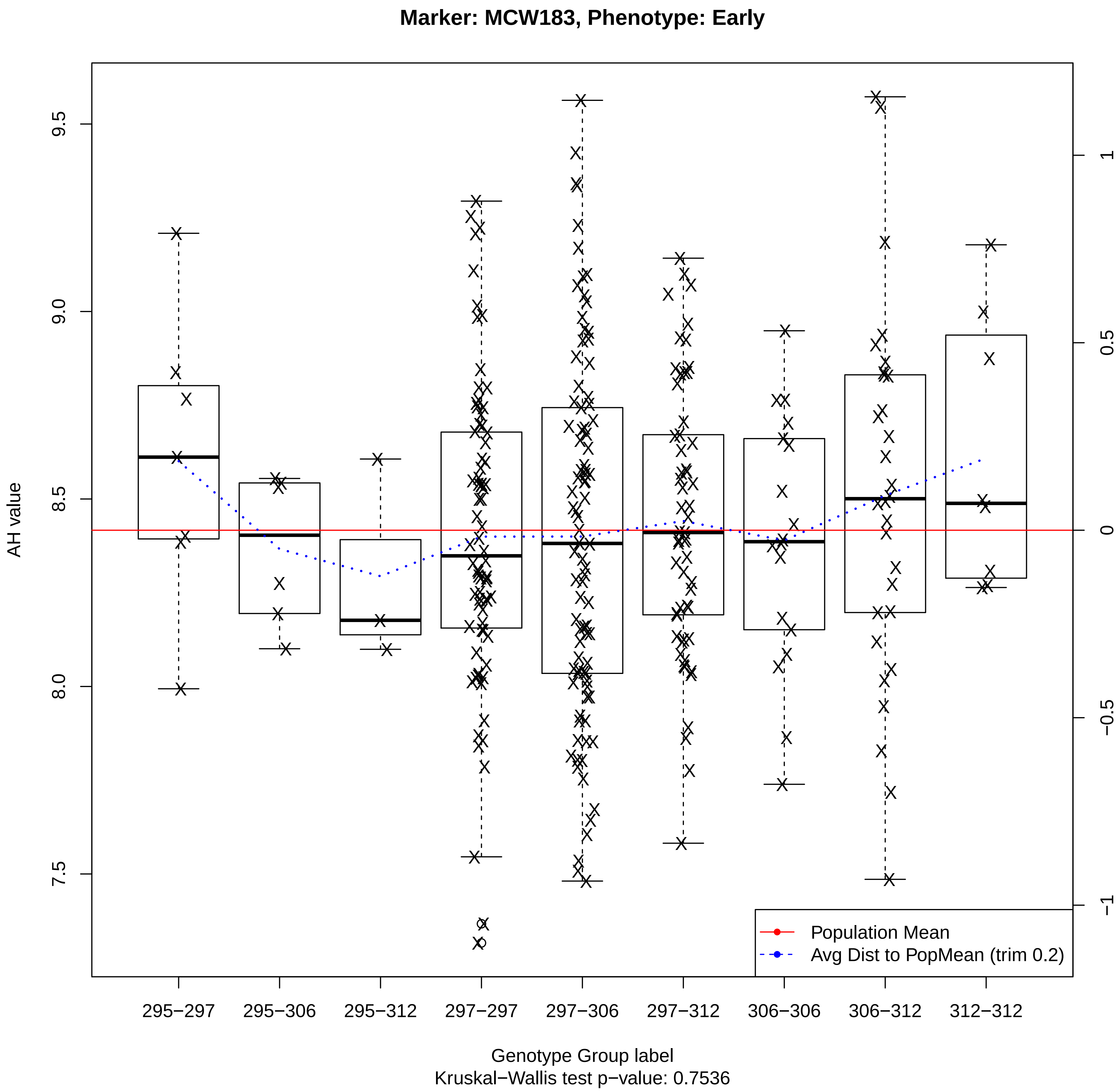

Marker: MCW183, Phenotype: Early
AH value
9.5
9.0
8.5
8.0
7.5
1
0.5
0
−0.5
−1
Population Mean
Avg Dist to PopMean (trim 0.2)
295−297
295−306
295−312
297−297
297−306
297−312
306−306
306−312
312−312
Genotype Group label
Kruskal−Wallis test p−value: 0.7536

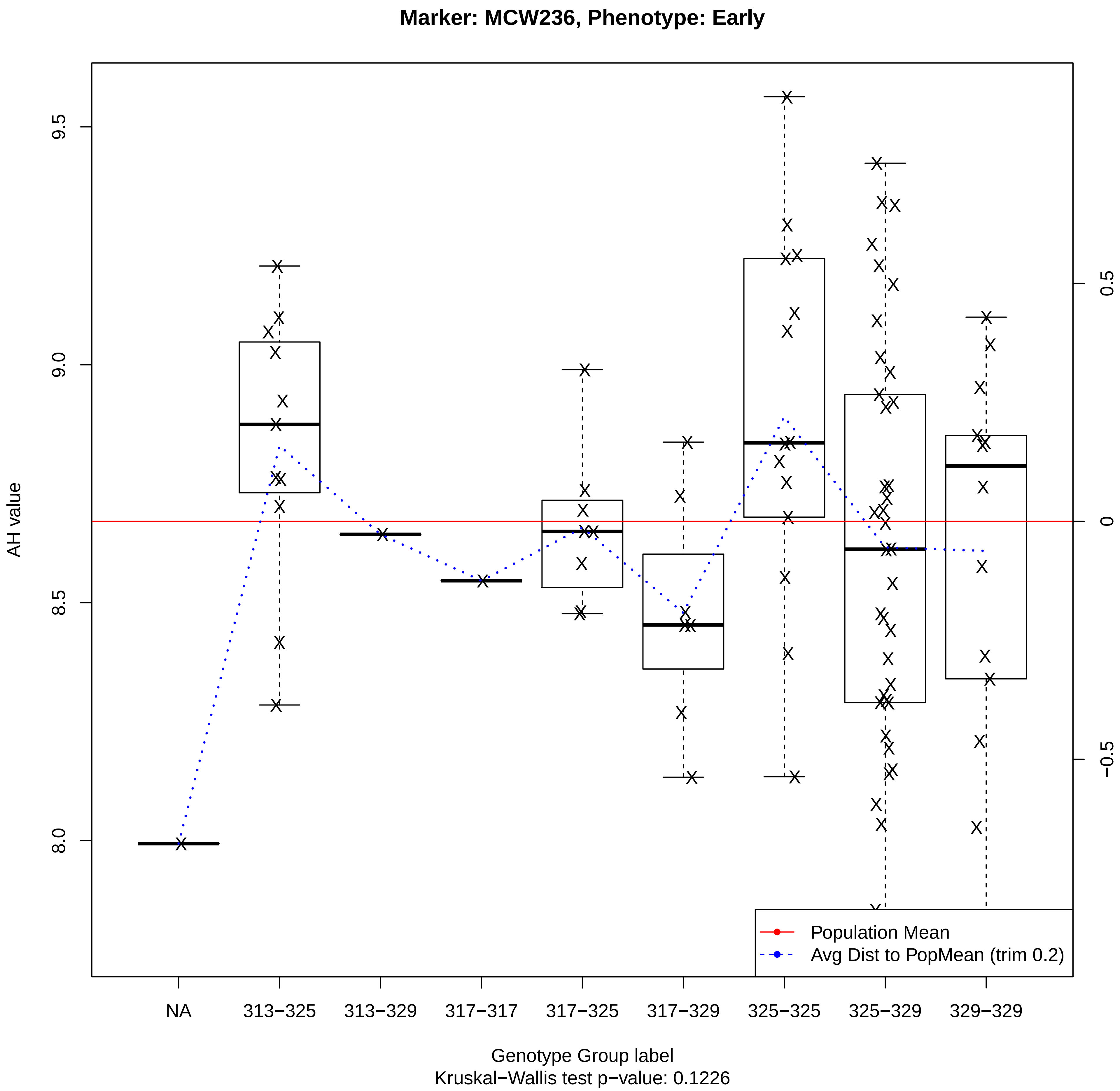

Marker: MCW236, Phenotype: Early
AH value
9.5
9.0
8.5
8.0
0.5
0
−0.5
Population Mean
Avg Dist to PopMean (trim 0.2)
NA
313−325
313−329
317−317
317−325
317−329
325−325
325−329
329−329
Genotype Group label
Kruskal−Wallis test p−value: 0.1226

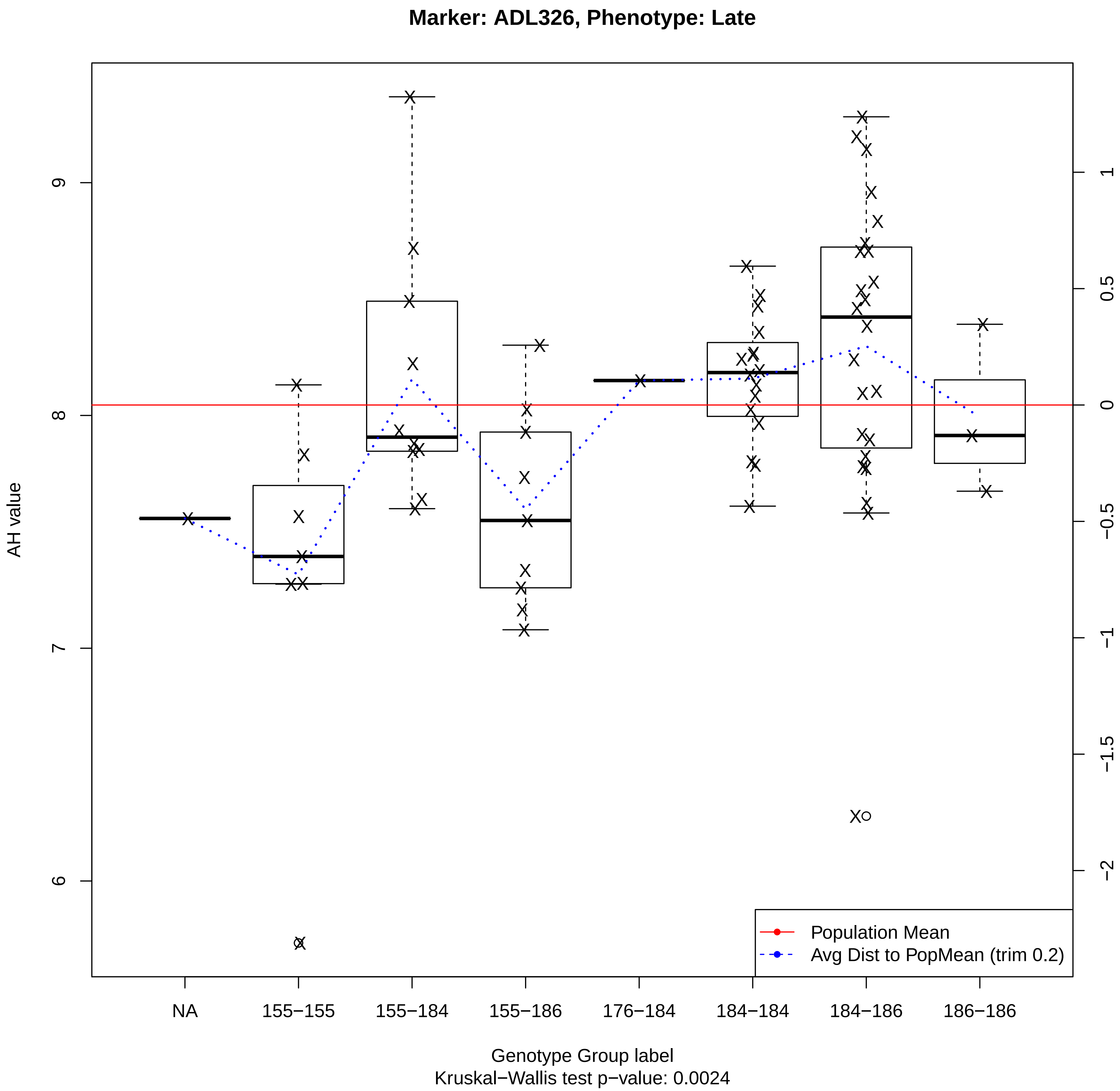

Marker: ADL326, Phenotype: Late
AH value
9
8
7
6
1
0.5
0
−0.5
−1
−1.5
−2
NA
155−155
155−184
155−186
176−184
184−184
184−186
186−186
Population Mean
Avg Dist to PopMean (trim 0.2)
Genotype Group label
Kruskal−Wallis test p−value: 0.0024

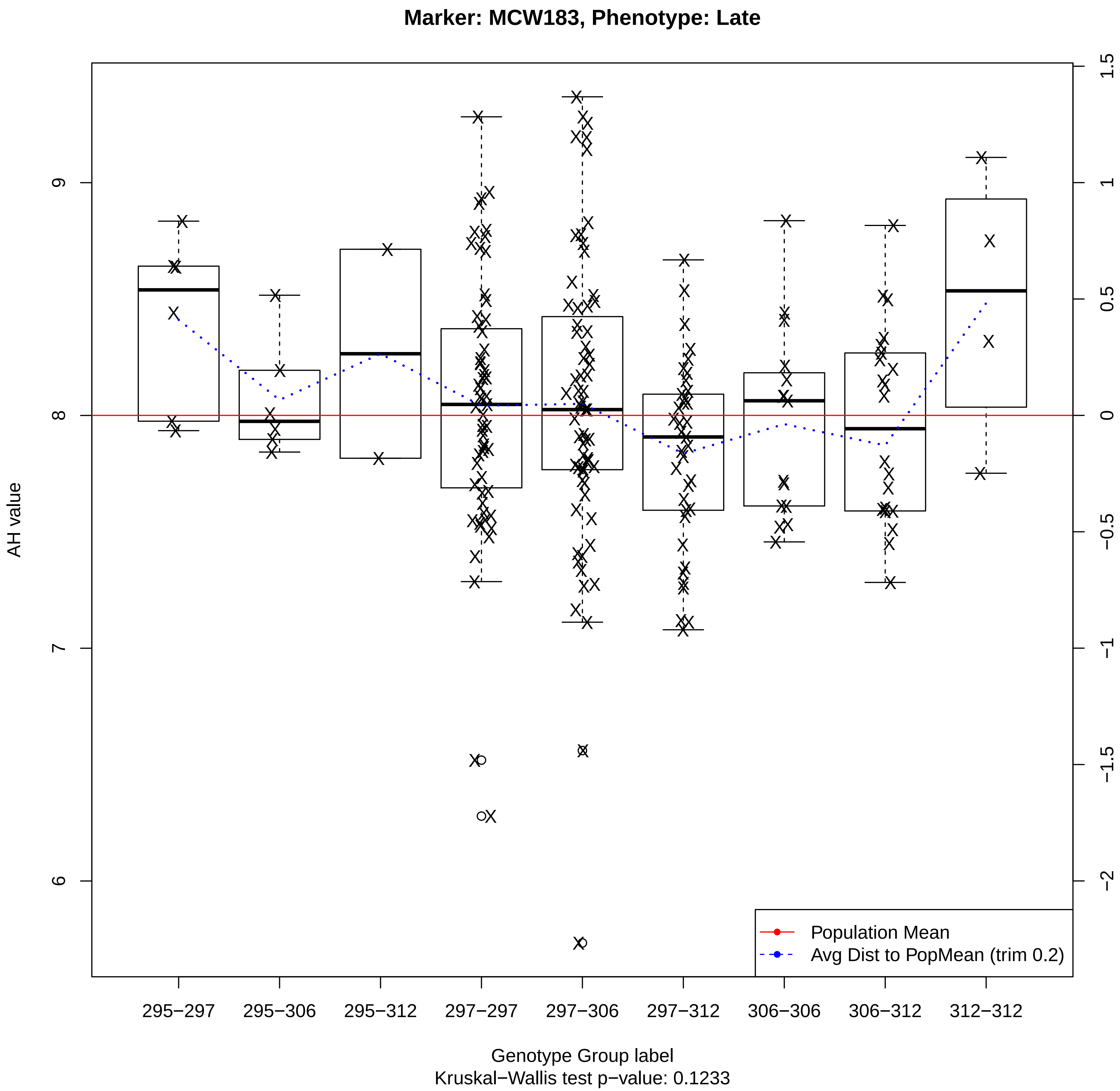

Marker: MCW183, Phenotype: Late
AH value
9
8
7
6
1.5
1
0.5
0
−0.5
−1
−1.5
−2
295−297
295−306
295−312
297−297
297−306
297−312
306−306
306−312
312−312
Population Mean
Avg Dist to PopMean (trim 0.2)
Genotype Group label
Kruskal−Wallis test p−value: 0.1233

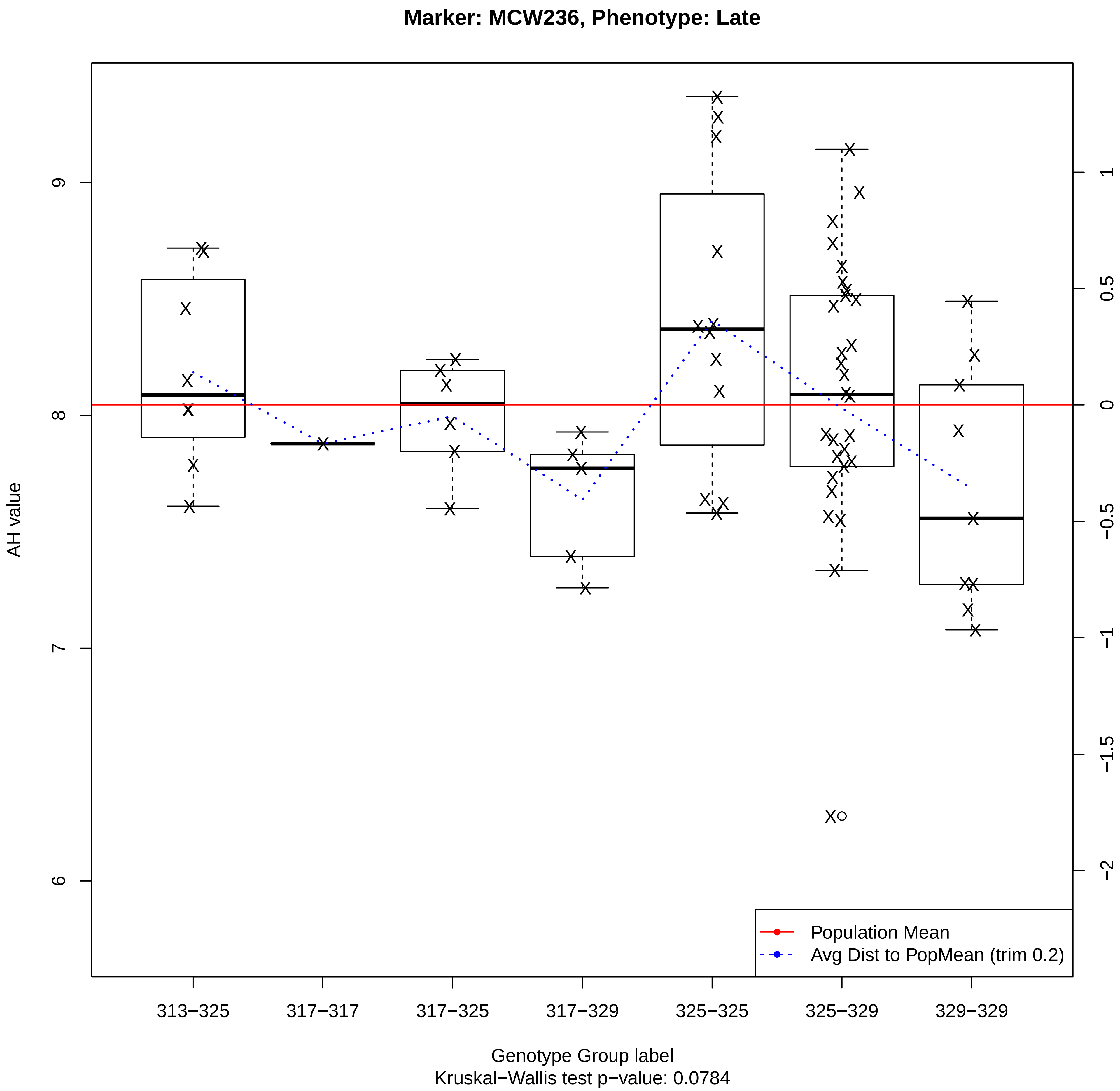

Marker: MCW236, Phenotype: Late
AH value
9
8
7
6
1
0.5
0
−0.5
−1
−1.5
−2
313−325
317−317
317−325
317−329
325−325
325−329
329−329
Population Mean
Avg Dist to PopMean (trim 0.2)
Genotype Group label
Kruskal−Wallis test p−value: 0.0784
